# A new mouse line with reduced GluA2 Q/R site RNA editing exhibits loss of dendritic spines, hippocampal CA1-neuron loss, learning and memory impairments and NMDA receptor-independent seizure vulnerability

**DOI:** 10.1186/s13041-020-0545-1

**Published:** 2020-02-27

**Authors:** Lyndsey M. Konen, Amanda L. Wright, Gordon A. Royle, Gary P. Morris, Benjamin K. Lau, Patrick W. Seow, Raphael Zinn, Luke T. Milham, Christopher W. Vaughan, Bryce Vissel

**Affiliations:** 1 Centre for Neuroscience and Regenerative Medicine (CNRM), Faculty of Science, University of Technology Sydney, PO Box 123 Broadway, Sydney, NSW 2007 Australia; 2 St Vincent’s Centre for Applied Medical Research, Sydney, 2011 Australia; 3 Department of Biomedical Sciences, Faculty of Medicine & Health Sciences, Macquarie University, Sydney, New South Wales 2109 Australia; 4 Middlemore Hospital, Counties Manukau DHB, Otahuhu, Auckland, 1062 New Zealand; 5 The University of Auckland, Faculty of Medical and Health Sciences, School of Medicine, Grafton, Auckland, 1023 New Zealand; 6 Kolling Institute of Medical Research, Royal North Shore Hospital, The University of Sydney, Sydney, 2065 Australia

**Keywords:** GluA2, AMPA receptors, RNA editing, Hippocampus, Seizures, Neurodegeneration, Alzheimer’s disease, Long term potentiation, Synapses

## Abstract

Calcium (Ca^2+^)-permeable AMPA receptors may, in certain circumstances, contribute to normal synaptic plasticity or to neurodegeneration. AMPA receptors are Ca^2+^-permeable if they lack the GluA2 subunit or if GluA2 is unedited at a single nucleic acid, known as the Q/R site. In this study, we examined mice engineered with a point mutation in the intronic editing complementary sequence (ECS) of the GluA2 gene, *Gria2*. Mice heterozygous for the ECS mutation (named GluA2^+/ECS(G)^) had a ~ 20% reduction in GluA2 RNA editing at the Q/R site. We conducted an initial phenotypic analysis of these mice, finding altered current-voltage relations (confirming expression of Ca^2+^-permeable AMPA receptors at the synapse). Anatomically, we observed a loss of hippocampal CA1 neurons, altered dendritic morphology and reductions in CA1 pyramidal cell spine density. Behaviourally, GluA2^+/ECS(G)^ mice exhibited reduced motor coordination, and learning and memory impairments. Notably, the mice also exhibited both NMDA receptor-independent long-term potentiation (LTP) and vulnerability to NMDA receptor-independent seizures. These NMDA receptor-independent seizures were rescued by the Ca^2+^-permeable AMPA receptor antagonist IEM-1460. In summary, unedited GluA2(Q) may have the potential to drive NMDA receptor-independent processes in brain function and disease. Our study provides an initial characterisation of a new mouse model for studying the role of unedited GluA2(Q) in synaptic and dendritic spine plasticity in disorders where unedited GluA2(Q), synapse loss, neurodegeneration, behavioural impairments and/or seizures are observed, such as ischemia, seizures and epilepsy, Huntington’s disease, amyotrophic lateral sclerosis, astrocytoma, cocaine seeking behaviour and Alzheimer’s disease.

## Introduction

Within the central nervous system (CNS), α-amino-3-hydroxy-5-methyl-4-isoxazolepropionic acid receptors (AMPARs) mediate the majority of fast excitatory neurotransmission [1]. AMPARs are tetrameric protein complexes composed of differing combinations of four subunits, denoted GluA1-GluA4 (also known as GluR1–4 and GluRA-D, for a full review of AMPAR architecture refer here [2]). Diversity of AMPARs is created through several mechanisms including differing subunit composition [3–6], posttranslational modifications [7, 8], alternative splicing [9] and a process known as RNA editing [10–12], all of which can profoundly alter AMPAR properties.

RNA editing at the Q/R site (position 607) of GluA2 is a crucial editing event occurring in AMPAR subunits [10, 13, 14]. At this site, an adenosine to inosine (A-to-I) editing event results in an exonically encoded CAG codon being edited to a CIG in the pre-mRNA [14]. The CIG codon is read as a CGG because the inosine, in most cases, is interpreted as a G during translation [15], resulting in a conversion of glutamine (CAG, i.e. Q) to an arginine (CGG, i.e. R). The editing-induced amino acid change has a profound impact: AMPARs containing edited GluA2(R) (which appear to constitute a majority of total AMPARs physiologically [4–6, 16]) are Ca^2+^-impermeable. This likely occurs because the arginine is positively charged and present in the pore-lining (M2) region [17] which, in contrast to the uncharged glutamine, prevents Ca^2+^flux. Alternatively, AMPARs that lack the GluA2 subunit (i.e. that are assembled from homomeric or heteromeric combinations of GluA1, A3 and A4), or that contain unedited GluA2(Q), are Ca^2+^-permeable [10, 18–22].

It is unclear why this GluA2 editing process has evolved [23–25], especially considering GluA2 Q/R site editing is ~ 99% efficient in the healthy adult brain. However, it is highly conserved [26, 27], hinting at a strong selective pressure for retaining the editing process [25, 28]. Although forced-edited mice expressing only GluA2(R) appear normal [23], suggesting unedited GluA2(Q) is not required for normal brain development and function, we have previously argued that unedited GluA2(Q) may have unrecognised physiological roles when present in mature AMPARs [10]. Furthermore, a decrease in editing efficiency has been observed in several human neurological conditions including Alzheimer’s disease [29–31], schizophrenia [30], Huntington’s disease [30], amyotrophic lateral sclerosis [32], astrocytoma [33], ischemia [34] as well as cocaine seeking behaviour in rats [35] (for a review see [36]). These findings suggest a decrease in GluA2 Q/R site editing efficiency (leading to increased proportions of unedited GluA2(Q) subunits, relative to edited GluA2(R)), may play a role in the aetiology of these conditions.

Supporting this hypothesis, several seminal studies have described the phenotype of mice with forced expression of varying levels of unedited GluA2(Q). In these studies, mice were genetically engineered with deletions of the *Gria2* intronic editing complimentary sequence (ECS) that is necessary for Q/R site RNA editing [37–39]. The mice exhibited severely compromised phenotypes including a propensity for seizures, premature mortality, synaptic transmission abnormalities and hippocampal cell death [37–39] (also see a study in zebrafish [40]). The seizures and premature mortality are reminiscent of adenosine deaminase acting on RNA 2 (ADAR2) knockout (KO) mice (ADAR2 is the enzyme responsible for editing GluA2 [41]). ADAR2 KO mice have a higher proportion of unedited GluA2(Q) compared with edited GluA2(R) and their phenotype can be significantly improved by the forced expression of edited GluA2(R), suggesting unedited GluA2(Q) is the primary driver of ADAR2 KO mouse abnormalities [28, 42]. Furthermore, the expression of unedited GluA2(Q) in adult mice renders hippocampal neurons more vulnerable to ischemic insult [34, 43, 44].

Collectively, these studies hint to possible roles for unedited GluA2(Q) in the aetiology of several neurological conditions, but there is much yet to learn and further studies are needed. In particular, the phenotype of mice genetically engineered to express higher proportions of unedited GluA2(Q) has not yet been fully characterised, in part because of the reduced lifespan of prior models, leading to a lack of understanding of the role of unedited GluA2(Q) in vivo. In this study we therefore generated a new mouse line with a single point mutation in the ECS that was previously found in vitro to regulate GluA2 Q/R site RNA editing [45]. We have named this model GluA2^+/ECS(G)^. By introducing a single point mutation, rather than removing the ECS entirely (as was done in prior models [37–39]), we aimed to generate a model with a more subtle phenotype that was amenable to long-term phenotyping. We herein report that these mice have reduced GluA2 Q/R site RNA editing and provide initial anatomical, behavioural, electrophysiological and seizure phenotyping, with a focus on the hippocampus. We suggest that the mice will be of value to the field for future studies investigating the role of unedited GluA2(Q) in physiology and disease.

## Materials and methods

### Generation of mice

**Fig. 1:**
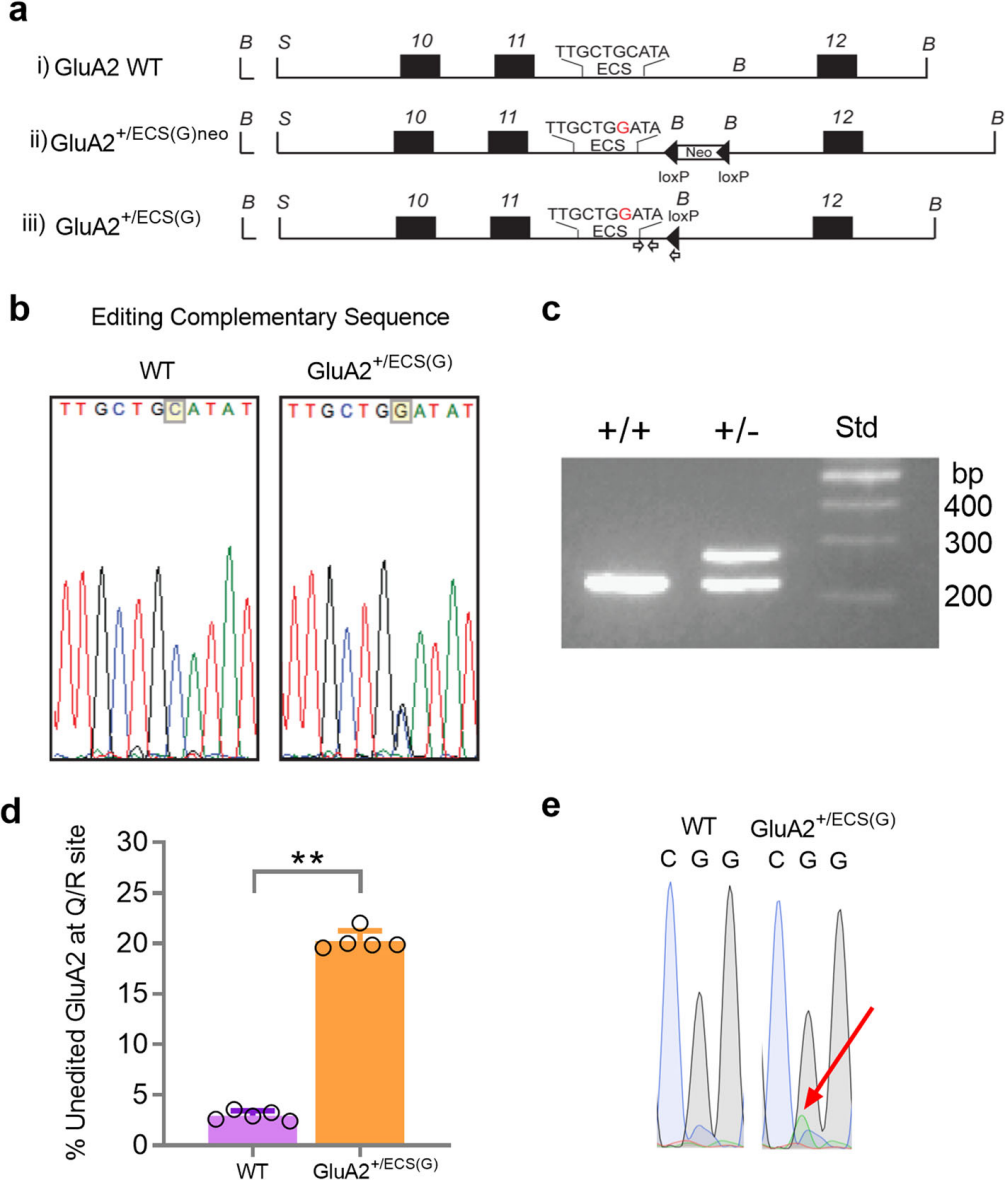
Generation of GluA2^+/ECS(G)^ mice and GluA2 Q/R site editing efficiency analysis. **a** Schematic representation of the i) GluA2 WT allele, ii) targeted GluA2^+/ECS(G)neo^ allele and iii) the targeted GluA2^+/ECS(G)^ allele, after the removal of the floxed neo cassette by Cre-mediated recombination. Exons 10, 11 and 12 are shown (black boxes). Black arrows indicate loxP sites. The position of the cytosine to guanine mutation within the ECS is indicated in red. White arrows indicate primer sets used for genotype analysis. **b** DNA sequencing of WT and GluA2^+/ECS(G)^ mice confirmed the single cytosine to guanine mutation in the ECS of heterozygous mice, as highlighted in yellow. **c** Genotype analysis of WT and GluA2^+/ECS(G)^ mice by PCR shows a band at 200 bp in WT and two bands at 200 bp and 250 bp in heterozygous mice. **d** GluA2^+/ECS(G)^ mice exhibit a significant increase in the proportion of unedited GluA2(Q) (*n* = 5/genotype; Mann-Whitney t-test). **e** Representative image of sequences from WT and GluA2^+/ECS(G)^ mice. The red arrow indicates the increased presence of A nucleotide indicating unedited RNA at the Q/R site of GluA2

A targeting construct, including exons 9–12 of the *Gria2* gene, was generated from DNA cloned from a 129S6 DNA genomic library (Fig. 1a). The final construct included a single base pair guanine to cytosine mutation within the ECS which altered the endogenous ECS sequence 5′-TTTGCTG**C**ATA-3′ to the mutated sequence 5′-TTTGCTG**G**ATA-3′. This particular nucleotide mutation was selected as it resulted in a significantly higher proportion of unedited GluA2 RNA in an in vitro study [45]. Additionally, a neomycin gene, surrounded by loxP sites, was placed downstream of the ECS, while a thymidine kinase (TK) gene was inserted at the 3′ end of the construct. The construct was electroporated into CCE embryonic stem cells, which originate from 129SvEv mice. Colonies resistant to G418 and ganciclovir were isolated. An ES cell colony that contained the desired mutant allele was identified. This ES cell colony was electroporated with a Cre-expressing plasmid and re-plated in the absence of G418 and ganciclovir, thus excising the neomycin and leaving a single loxP site. Resulting ES cell colonies containing the neomycin-deleted allele were chosen for blastocyst injection into C57B6 embryos. Chimeric mice were bred to 129S6 mice and offspring containing the mutant allele were subsequently maintained in a 129S6 background. Mutant mice were designated GluA2^+/ECS(G)^. In all experiments, both heterozygous male and female mice were used and compared with wildtype (WT) littermate controls aged 8–10 weeks and experiments were performed blind to genotype. Some experiments were conducted with 36-week-old mice, as indicated in the manuscript. The same mice were used for open field, rotarod and fear conditioning, in that order. Mice used in electrophysiology experiments were behaviorally naïve.

### Genotyping

PCR of genomic DNA from tail biopsies was conducted for genotyping analysis. PCR was routinely performed with oligonucleotide primers for the *Gria2* wild-type allele (Forward: 5′-GTG TCT CTT GGG GAA GTT CAA T-3′, and Reverse: 5′- TGA TAT ATT TCC CCT CTT CTC AGC − 3′). For the targeted allele, a primer was designed from within the loxP sequence with Reverse: 5′-TGC CCA CAT CTA AGA TTG TTG GAC-3′). PCR product sizes for the wild-type and targeted allele were 200 bp and 250 bp, respectively.

### DNA sequencing

A single-step multiplex PCR targeted at amplifying exon 11 of *Gria2* was utilized for confirmation of the mutation to the ECS. (Forward: 5′-TGG CAC ACT GAG GAA TTT GA-3′ and Reverse: 5′- TCA CAA ACA CAC CCA TTT CCA-3′). The PCR assay was carried out in a final volume of 50 μl containing 1 x Reaction buffer, 200 μM dNTPs, 0.5 μM of each primer, 0.01 U of Q5 Hot start High Fidelity DNA Polymerase (New England Biolabs) and 1 μL of DNA template. PCR products were purified using Qiaquick PCR purification kit (Qiagen). DNA sequencing was performed using an ABI 3130xl Genetic Analyzer (Applied Biosystems) with Big Dye 3.0 chemistry, after which sequences were edited and assembled using Finch TV (Geospiza Inc.).

### RNA editing assay with sanger sequencing

Animals were anesthetized with isoflurane, brains were rapidly dissected, and the hippocampus was isolated, snap frozen (in dry ice) and stored at − 80 °C until required. Total RNA was isolated using a Maxwell® RSC simplyRNA Tissue Kit (Promega, Cat# AS1340) and a Maxwell® RSC Instrument (Promega), according to the manufacturer’s instructions. As part of the protocol a DNAse treatment step was performed. cDNA was synthesized using SuperScript III (Invitrogen) and RNAseH (Invitrogen) in a total volume of 20 μl. Both no-reverse transcriptase and master mix controls were included to ensure no contamination or cross-contamination was present in the samples. PCR amplification was performed across the editing region of GluA2 using the cDNA template (Forward: 5′- CAGCAGATTTAGCCCCTACG − 3′ and Reverse: 5′- AGCCGTGTAGGAGGAGATGA − 3′), amplifying a 226 bp product. PCR products were run on a 2% agarose gel and bands were excised and purified using a QIAquick Gel Extraction Kit (Qiagen), according to the manufacturer’s instructions. 20 ng of purified DNA was dried with 3.2 pmol of the forward primer. Samples were sequenced at Garvan Molecular Genomics using an ABI 3130XL Genetic Analyser and were visualised using SnapGene Viewer. The percentage of unedited RNA was quantified by measuring the peak height of the A nucleotide at the Q/R site of GluA2 sequences relative to the peak height of the G nucleotide at this position using the formula: percentage unedited templates = (peak height A/(peak height A + peak height G)) × 100, as previously published [41, 46]. The peak heights were calculated using Image J (NIH).

### *BbvI* RNA editing assay

Animals were anesthetized with isoflurane, brains were rapidly dissected and the hippocampus was isolated, snap frozen with isopentane and dry ice and stored at − 80 °C until analysis. Total RNA was isolated using Trizol Reagent (Invitrogen) according to the manufacture’s protocol and subjected to DNAse treatment (Invitrogen). cDNA was synthesized using SuperScript III (Invitrogen) and RNAse-H (Invitrogen) in a total volume of 20 μl. PCR amplification was performed across the editing region of GluA2 using the cDNA template (Forward: 5′-TTC CTG GTC AGC AGA TTT AGC C-3′ and Reverse: 5′-AGA TCC TCA GCA CTT TCG-3′). PCR products were run on a 1.8% agarose gel and the bands were excised and gel purified using the QIAquick Gel Extraction Kit (Qiagen), yielding 30 μl of product. The gel-purified products were digested with 1 U of *BbvI* enzyme (New England Biolabs) in a total volume of 20 μl for a total of six h at 37 °C. The reaction was terminated at 65 °C for 20 min. The products were run on 10% TBE gels (Invitrogen). The bands were quantified using Image J and expressed as a percentage of the unedited band (81 bp) divided by the unedited band (81 bp) + the edited band (68 bp).

### Kainic acid-induced seizure activity

8–10-week-old GluA2^+/ECS(G)^ mice and littermate controls were intraperitoneally injected with 10 mg/kg kainic acid (KA, Sigma) and were observed for 1 h following injection. This dose was insufficient to induce seizures in WT mice, but was sufficient to induce mild to moderate seizures in GluA2^+/ECS(G)^ mice. Where stated, mice were injected immediately prior to KA administration with AP-5 (20 mg/kg; Tocris) or IEM-1460 (7.5 mg/kg; Tocris).

Seizure stage was assessed by the maximum score within a five minute window, according to a modification of the Racine scale: stage 0 - normal behaviour; stage 1 - immobility; stage 2 - rigidity, whisker twitching; stage 3 - forelimb pawing, head bobbing and tail whipping; stage 4 - intermittent rearing and falling with forelimb/jaw clonus; stage 5 - continuous rearing and falling > 30 s; stage 6 - generalized tonic-clonic seizures with whole body convulsions; stage 7 death.

### Electrophysiology

All tissue used for electrophysiology experiments was derived from behaviourally naïve animals. Coronal CA1 slices (400 μm) were prepared using a vibratome (VT1000S; Leica Microsystems) in ice-cold artificial cerebrospinal fluid (ACSF) of composition: 126 mM NaCl, 2.5 mM KCl, 1.4 mM NaH_2_PO_4_, 1.2 mM MgCl_2_, 2.4 mM CaCl_2_, 11 mM glucose and 25 mM NaHCO_3_. Slices were maintained (≥ 1.5 h) at 30–32 °C in a submerged chamber containing carbogen equilibrated (95% O_2_, 5% CO_2_) ACSF before being individually transferred to a recording chamber (≥ 30 min prior to recording) and superfused continuously (2.5 ml.min^− 1^) with carbogen equilibrated ACSF using a recirculation system. A glass bipolar stimulating microelectrode (2–3 MΩ, filled with ACSF) was placed in the stratum radiatum.

For patch clamp experiments, CA1 neurons were visually identified using Dodt-tube optics on an upright microscope (Olympus BX51). Whole-cell voltage-clamp recordings were conducted via an Axopatch 700B patch clamp amplifier, using an internal solution of the following composition: 125 mM CsMeSO_3_, 10 mM CsCl, 5 mM HEPES, 0.4 mM EGTA, 4 mM NaCl, 1 mM MgCl_2_, 2 mM MgATP, 0.3 mM NaGTP, 3 mM QX314 and 0.1 mM spermine (pH = 7.3; osmolarity = 280–285 mOsM). Series resistance (< 25 MΩ) was compensated by 80% and continuously monitored during experiments. Liquid junction potentials of − 15 mV were corrected. Electrically evoked AMPA-receptor mediated excitatory postsynaptic currents (EPSCs), obtained in the presence of the GABA_A_-receptor blocker picrotoxin (100 μM) and the NMDAR antagonist DL-AP5 (50 μM), were elicited once per 12 s. 1-naphthyl acetyl spermine (Naspm, 50 μM) was bath applied to block Ca^2+^-permeable AMPARs.

For long-term potentiation (LTP) experiments, field excitatory postsynaptic potentials (fEPSPs) were recorded via a glass microelectrode (2–3 MΩ, filled with ACSF) placed in the stratum radiatum 300–400 μm from the stimulating electrode. fEPSPs were evoked once every 30 s, at an intensity adjusted to produce fEPSPs with amplitudes corresponding to ~ 50% of maximal responses. After obtaining 20 min of stable baseline fEPSPs, three trains of high-frequency stimulation (HFS, 100 pulses at 100 Hz, inter-train interval of 10 s) to induce LTP, and fEPSPs were recorded for another 60 min.

### Golgi staining

Mice were anesthetized with isoflurane and cervically dislocated. Brains were stained using the FD Rapid GolgiStain Kit (FD NeuroTechnologies) as per the manufacturer’s recommendations. To analyse dendritic morphology, Golgi-stained CA1 neurons were manually traced at 100x magnification with Neurolucida (MBF Bioscience) and total dendritic lengths were measured and quantified using Neurolucida Explorer. Scholl analysis was performed with Neurolucida Explorer to demonstrate the branching patterns of neuronal dendritic trees. Spine density was assessed by counting the number of spines in 3 branches per neuron of branch orders 2–4. All protrusions no longer than 2 μm were counted as spines if they were continuous with the dendritic shaft. The spine density was defined as the number of spines on 10 μm of dendritic length.

### Immunohistochemistry

Immunohistochemistry was conducted as previously described [47]. Tissue was cryosectioned at 40 μm (Leica Microsystems). Free-floating sections were incubated in mouse anti-NeuN (1:500; Merck Millipore) for 72 h followed by overnight incubation in biotin-labeled chicken anti-mouse secondary antibody (1:250; Invitrogen). Immunolabeling was detected using HRP-labeled avidin-biotin complex and 3.3′-Diaminobenzidine substrate (DAB; Vector Laboratories).

### Stereology

Design based stereology, using with Stereo Investigator 7 (MBF Bioscience), was used to estimate cell populations, as previously described [47]. Briefly, estimates were conducted on the dorsal hippocampus at the anteroposterior (AP) positions from between Bregma − 1.34 mm and − 2.3 mm. For neuronal population estimates, a minimum 20 sampling sites were sampled per section on a grid size of 84 μm × 60 μm and a counting frame size of 30 μm × 30 μm. For all cell population estimates, a guard zone of 5 μm and a dissector height of 10 μm were used. Each marker was assessed at one in every sixth section, with a total of five sections being sampled. Both the CA3 and CA1 regions of the hippocampus were sampled.

### Cobalt uptake

Mice were sacrificed, the brain rapidly removed and coronal sections (400 μm) were cut with a vibratome (Leica Microsystems) in ice cold Krebs solution buffer containing (in mM: 125 NaCl, 2.5 KCl, 26 NaHCO_3_, 1.25 NaH_2_PO_4_, 25 glucose, 2 CaCl_2_, 1 MgCl_2_) bubbled with 95% O_2_/5% CO_2_. Slices were allowed to recover for 1 h in Krebs at 28 **°**C. Slices were transferred into a pre-stimulation solution of low-sodium, low-calcium Krebs solution containing (in mM: 50 NaCl, 2.5 KCl, 26 NaHCO_3_, 1.25 NaH_2_PO_4_, 25 glucose, 0.5 CaCl_2_, 2 MgCl_2_,) with 0.5 μM TTX (Tocris) and 100 μM AP-5 (Tocris). Control slices were pre-treated with Krebs containing NBQX (20 μM; Tocris) or GYKI (100 μM; Sigma). Slices were stimulated with kainate (20 μM; Sigma) in the low-sodium, low-calcium Krebs solution, with the addition of CoCl_2_ (1.5 mM). Slices were washed in Kreb’s solution without divalent ions (in mM: 50 NaCl, 2.5 KCl, 26 NaHCO_3_, 1.25 NaH_2_PO_4_, 25 glucose) containing EDTA (0.5 mM) for 10 min, before being incubated in Kreb’s solution without divalent ions containing 0.12% NH_4_S for 5 min to precipitate intracellular Co^2+^. Slices were then washed with Kreb’s solution without divalent ions for 5 min and fixed in 4% paraformaldehyde overnight and equilibrated in 30% sucrose for three days. Sections were cut in OCT (Scigen) at 40 μm on a cryostat (Leica Microsystems) and mounted onto gelatin-coated slides (Thermo Fisher Scientific).

For silver intensification, sections were incubated in 2% Na_2_WO_4_ for 10 min and then incubated in developer solution (8 parts of AgNO_3_ solution: 1% Triton X-100, 7.5% CH_3_COOH, 30.3 mM Na-acetate, 2.94 mM AgNO_3_; 1 part of 5% Na_2_WO_4_; 1 part of 0.25% ascorbic acid) in the dark for 15 min and washed in 2% Na_2_WO_4_, dried, rinsed in 100% xylol, and coverslipped.

### Co-Immunoprecipitations

Co-immunoprecipitation experiments were conducted as previously described [16, 48, 49], with modification. Mice were sacrificed, brains were rapidly dissected, and the hippocampus was isolated, frozen with dry ice and stored at − 80 °C until required. Tissue was homogenized in 50 mM Tris-HCl (pH 7.4) with protease inhibitors (Sigma). Cell membranes were sedimented by centrifugation at 100,000 x g for 30 min at 4 °C. The pellet was resuspended in 750 μl of 1% Triton X-100, 50 mM Tris-HCl (pH 7.4) and 1 mM EDTA followed by incubation for 45 min at 37 °C. For co-immunoprecipitations, 3 μg of antibody (GluA1, GluA2, GluA2/3, GluA4, GluA1 + 2/3, GluA2/3 + 4 or IgG) was incubated in 50 μL (1.5 mg) of Dynabead protein A (Invitrogen) according to manufacturer’s instructions. 105 μl of sample protein was incubated with antibody-bound beads at RT for 30 min with gentle agitation. Protein was subjected to two rounds of immunoprecipitations. Bound fractions from the first round of immunoprecipitation were eluted using the provided elution buffer. LDS sample buffer and reducing agent (Invitrogen) were added to bound and unbound fraction samples followed by incubation for 10 min at 70 °C prior to SDS PAGE and immunoblotting. The following AMPAR subunit-specific antibodies were used for Western blotting: GluA1 (1:1000, Millipore), GluA2/3 (1:1000, Millipore), GluA2 (1:1000, Millipore) and GluA3 (1:1000, Cell Signaling). The percent of total AMPAR subunit remaining in the unbound fraction was calculated based on the standard curve created from control IgG immunoprecipitated tissue.

### Open field test

The open field test (OFT) was conducted as previously described [47]. Briefly, mice were placed in an arena (40x40cm) enclosed with clear plexiglass walls that was situated in a large sound attenuating cubicle. Mice were placed into the center of the arena and allowed to explore the test box for 10 min, while a computer software program (Activity Monitor; Med Associates) recorded activity via photobeam detection inside the testing chambers. The total distance traveled over the course of the 10 min was recorded.

### Rotarod

Mice were placed on the suspended beam of the rotarod facing away from the viewer for 5 min. The rotarod was started once all mice were placed on the beams and rotated at a rate of 4 rpm which increased to 40 rpm over the course of 5 min. Animals were taken off the rotarod once they fell to the catch tray below or after 5 min had elapsed. The total time spent on the beam was recorded. Animals were exposed to the test once a day for three consecutive days.

### Fear conditioning

Contextual fear conditioning was conducted as previously described [47]. Briefly, training and testing took place in cube-shaped fear-conditioning chambers (32 × 27 × 26 cm; Med Associates Inc.) that had a clear plexiglass door, ceiling and rear wall and grey aluminum side walls. Each chamber had a removable grid floor, which consisted of 36 parallel rods spaced 8 mm apart. Positioned under the grid was a removable aluminum tray for collection of waste. The rods were connected to a shock generating and scrambling system, which delivered a current to elicit a foot shock. This system was connected to and controlled by computer software (FreezeFrame2, Actimetrics). A video camera, which was positioned in front of the chambers, recorded the behavior of the mice during training and testing. On the conditioning day, mice were placed into a fear-conditioning chamber in which the environment (context) was controlled. Mice were allowed time (3 min) to explore the context freely, prior to receiving a single moderate footshock (0.5 mA, 2 s). Following shock, all mice remained in the chamber for 30 s and were then immediately returned to their homecages. The following day, the mice were re-exposed to the same context and behavior was recorded for 3 min. Freezing was assessed as a measure of fear on all days using a 4 s sampling method by investigators, who were blind to the genotype. The number of observed freezes was averaged and divided by the total number of samples taken to yield a percentage of freezing. Data is presented as the average percentage of freezing during the 3 min period prior to shock delivery on conditioning day and during the 3 min test period on testing day.

### Statistics

All statistical analysis was performed using GraphPad Prism Version 7.0 (GraphPad Software, Inc). For seizure analysis, scores were square root transformed to produce a normal distribution. Data sets were tested for outliers using a Grubbs’ test. Differences between means were assessed, as appropriate, by t-tests or one/two-way ANOVAs (with or without repeated measures, followed by Bonferroni *post-hoc* analysis). Where repeated measures ANOVAs were performed, we used the Geisser-Greenhouse correction (i.e. did not assume sphericity). For t-tests, data sets were first tested for normality (the D’Agostino & Pearson test where possible, or the Shapiro-Wilk test if n’s were too small for the D’Agostino & Pearson test), before using either parametric or non-parametric tests, as appropriate. For parametric tests, an F test for variance, calculated in GraphPad Prism Version 7.0, was used to determine whether standard deviations were equal between groups. If they were unequal, Welch’s correction was applied to the t test. For non-parametric tests, the Mann-Whitney test was used. Statistical significance was defined at *p < 0.05* and presented as *p < 0.05, **p < 0.01, ***p < 0.001 and ****p < 0.0001. Results are displayed as mean ± standard deviation, unless otherwise indicated.

## Results

### GluA2^+/ECS(G)^ have ~ 20% unedited GluA2(Q) RNA

A prior in vitro study identified several key residues in the intronic ECS that are necessary for maintaining the efficiency of GluA2 pre-mRNA Q/R site editing [45]. In particular, when mutating a cytosine sitting within the ECS of intron 11 to a guanine (see mutant *B13M3* in [45]), Higuchi et al., found < 1% of GluA2 transcripts were edited. Building off this observation, we designed a transgenic mouse in which the ECS was altered from the endogenous sequence 5′-TTGCTG**C**ATA-3′ (Fig. 1)a(i), to the sequence 5′-TTGCTG**G**ATA-3′.

Mice expressing the final mutant allele termed GluA2^+/ECS(G)^ (Fig. 1a(iii)) were maintained as heterozygotes. Homozygote mutants were not viable. DNA sequencing confirmed a guanine residue (G) in the ECS of heterozygous GluA2^+/ECS(G)^ mice, in the position a cytosine (C) residue would otherwise occur in the WT allele (Fig. 1b). DNA sequencing confirmed no alteration to the Q/R site in the DNA of WT and GluA2^+/ECS(G)^ mice (Additional file 1a). Heterozygous mice were identified by PCR of the downstream intronic loxP sequence (Fig. 1c).

By sequencing mRNA transcripts, we determined the efficiency of GluA2 RNA editing at the Q/R site (Fig. 1d-e). We found 2.95 ± 0.48% of GluA2 is unedited at the Q/R site in the hippocampus of WT mice (Fig. 1d). In contrast, GluA2^+/ECS(G)^ mice showed a significant increase in proportion of unedited GluA2(Q) RNA (20.3 ± 1.0% of total GluA2 transcripts) in the hippocampus (Fig. 1d; mean ± SD, *p = 0.0079* (Mann-Whitney test)), confirming the critical importance of the ECS sequence for regulating normal GluA2 RNA editing in vivo. Editing assays conducted via Sanger sequencing may yield higher than expected editing rates in WT mice (editing rates at the Q/R site of GluA2 are thought to be > 99% in the adult brain [50]). Using a separate cohort of GluA2^+/ECS(G)^ mice we therefore conducted a second RNA editing assessment using a standard *BbvI* restriction enzyme-based assay. This assay confirmed the significant increase in the proportion of unedited GluA2(Q) RNA transcripts in GluA2^+/ECS(G)^ mice (Additional file 1b-d). Q/R site RNA alterations were confirmed via RNA sequencing in WT and GluA2^+/ECS(G)^ mice (Fig. 1e and Additional file 1a). We also found the editing changes did not appear to grossly affect AMPAR subunit assembly (Additional file 2).

### GluA2^+/ECS(G)^ have decreased body weight, premature mortality and increased seizure susceptibility that is NMDA receptor independent

**Fig. 2:**
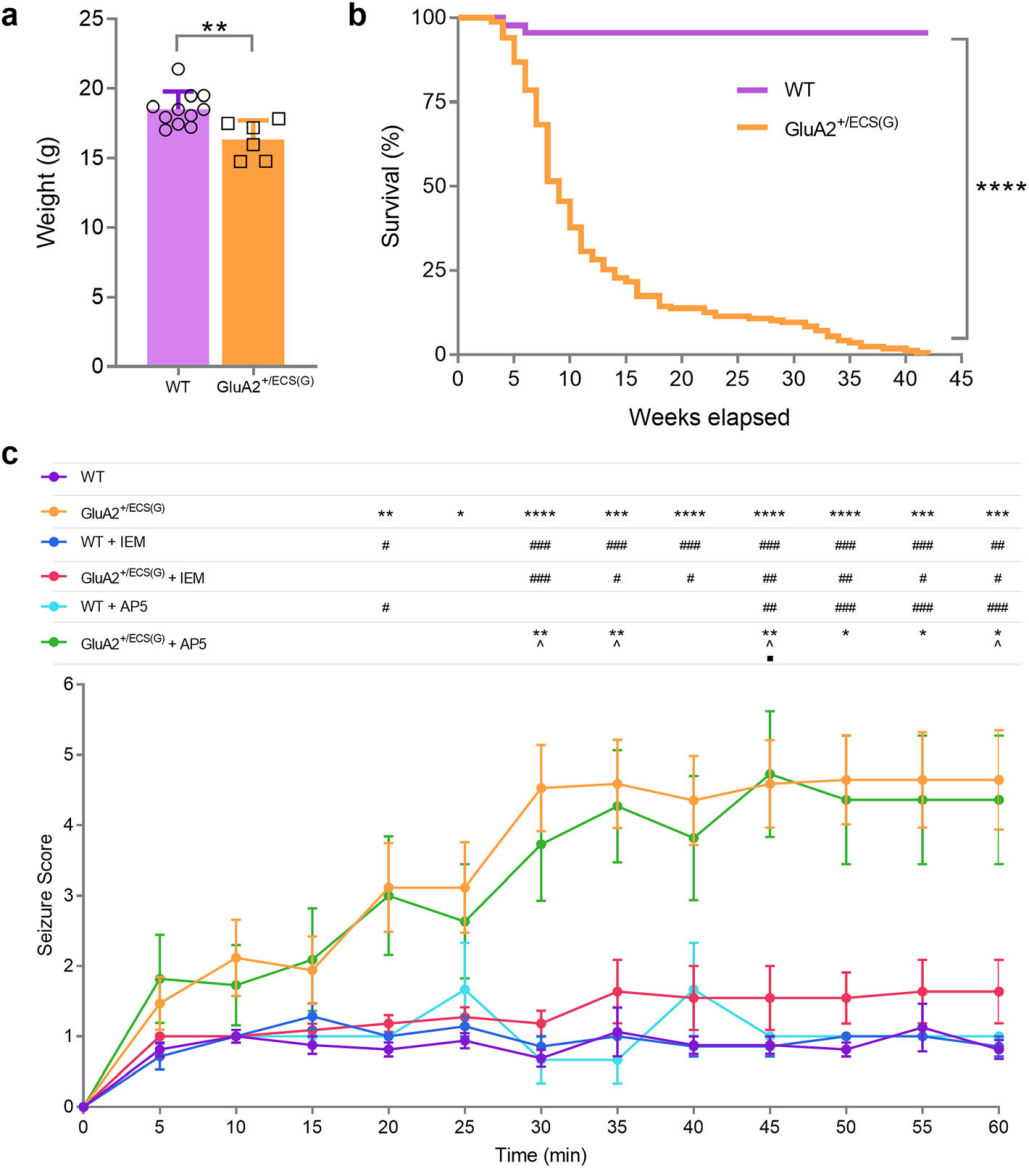
Body weight, survival curve and seizure susceptibility analysis of GluA2^+/ECS(G)^ mice. **a** GluA2^+/ECS(G)^ mice exhibit reduced body weight, as compared to WT littermates at 8 weeks of age (*n* = 6 GluA2 GluA2^+/ECS(G)^ mice, 11 WT; unpaired t-test). **b** GluA2^+/ECS(G)^ mice exhibit premature death and an approximate median survival age of 9 weeks (*n* = 42 WT, 170 GluA2^+/ECS(G)^ mice; Kaplan-Meier survival analysis). **c** GluA2^+/ECS(G)^ mice exhibit increased seizures following low-dose (10 mg/kg) of intraperitoneal KA injection that is blocked by the Ca^2+^-permeable AMPAR antagonist, IEM-1460 though not by AP5 (*n* = 16 (WT), 17 (GluA2^+/ECS(G)^), 7 (WT + IEM-1460), 11 (GluA2^+/ECS(G)^ + IEM-1460), 3 (WT + AP5), 11 (GluA2^+/ECS(G)^ + AP5); Repeated measures ANOVA). Data in (**a**) represent mean ± SD and in (**c**) represent mean ± SEM. * = compared to WT, # = compared with GluA2^+/ECS(G)^, ^ = compared with WT + IEM-1460, ■ = compared with WT + AP5. One symbol, *p < 0.05*, two symbols, *p < 0.01*, three symbols, *p < 0.001*, four symbols, *p* < 0.0001

Mice with reduced GluA2 Q/R site RNA editing have previously been shown to exhibit decreased body weight [38] and premature mortality, compared with WT littermates [37, 38]. In this study, we found that GluA2^+/ECS(G)^ mice were outwardly normal at birth, though they exhibited significant reductions in body weight at 8 weeks of age (Fig. 2a; mean ± SD, t = 3.239, df = 15, *p = 0.0046* (unpaired t-test)). A Kaplan-Meir survival curve (170 GluA2^+/ECS(G)^ and 42 WT littermates) revealed GluA2^+/ECS(G)^ mice were significantly susceptible to premature death as compared to WT littermates (Fig. 2b; χ2 = 77.07, df = 1, *p < 0.0001*), with a median survival of 9 weeks. Premature mortality in GluA2^+/ECS(G)^ mice was possibly due to spontaneous seizures, which were also observed.

The extended survival of GluA2^+/ECS(G)^, in comparison to previous mice with similar or greater GluA2 Q/R editing deficits [37, 38] allowed for an assessment of seizure susceptibility in this model and, in particular, whether seizure vulnerability is NMDAR-dependent. We injected the excitotoxin, kainic acid (KA), or KA plus the Ca^2+^-permeable AMPAR antagonist, IEM-1460, intraperitoneally in GluA2^+/ECS(G)^ and WT mice. GluA2^+/ECS(G)^ mice exhibited significant seizure activity following a relatively low dose of KA (10 mg/kg), indicating enhanced neuronal excitability in these mice (Fig. 2c; interaction F_(60,708)_ = 4.097, *p < 0.0001*, genotype and treatment F_(5,59)_ = 10.03, *p < 0.0001*, time F_(5.4, 319.3)_ = 43.22, *p < 0.0001* (repeated measures two-way ANOVA of square root transformed seizure scores, followed by Bonferroni *post-hoc* analysis). Furthermore, GluA2^+/ECS(G)^ mice injected with IEM-1460, a specific inhibitor of Ca^2+^-permeable AMPARs (that are either GluA2 lacking, or containing unedited Q/R site GluA2 [51]), exhibited reduced seizure behaviour that did not significantly differ from WT mice (*p > 0.05*). In contrast, injection of the NMDAR-antagonist, AP5, did not reduce seizure vulnerability. Remarkably, the observed seizures in GluA2^+/ECS(G)^ therefore appear to be NMDAR-independent. Combined, these results suggest that seizure susceptibility is due to the activation of Ca^2+^-permeable AMPARs in GluA2^+/ECS(G)^ mice.

### GluA2^+/ECS(G)^ have inwardly rectifying currents and enhanced LTP which is AMPAR-dependent

Ca^2+^-permeable AMPARs show inwardly rectifying current/voltage (I/V) relationships [52, 53]. We therefore sought to establish whether GluA2^+/ECS(G)^ mice exhibited this phenotype. Previous mouse models with reduced Q/R site RNA editing have illustrated altered AMPAR hippocampal CA1 current rectification and calcium permeability [37, 38]. It has generally been thought this is due to a reduction in the availability of edited GluA2, or of total GluA2 expression (i.e. an increased proportion of GluA2 lacking, Ca^2+^ permeable AMPARs [37, 38]). However, this could also be due to an increase in the proportion of AMPARs containing unedited GluA2.

**Fig. 3:**
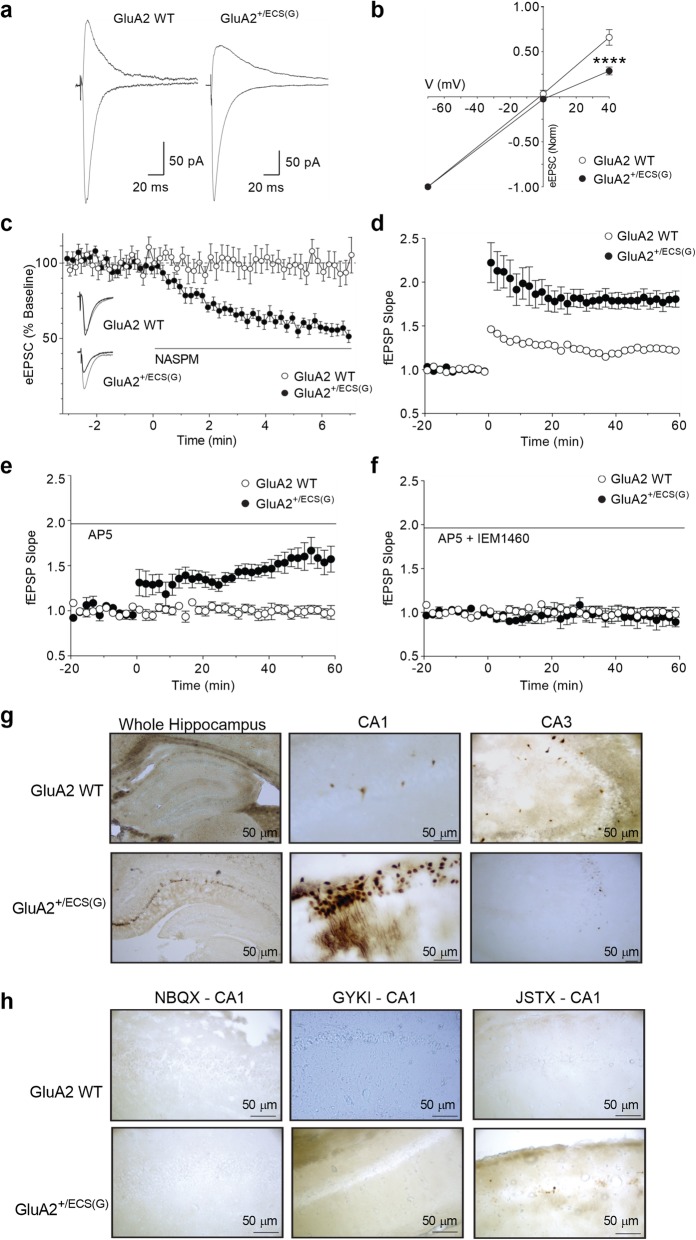
AMPAR-mediated excitatory synaptic transmission and long-term synaptic plasticity in CA1 hippocampal neurons. **a** Averaged traces of AMPA evoked EPSCs at − 70 and + 40 mV in WT and GluA2^+/ECS(G)^ mice. **b** Current-voltage (I/V) relationship of synaptic responses at − 70, 0 and + 40 mV in WT and GluA2^+/ECS(G)^ mice (*n*=10 GluA2^+/ECS(G)^ and 15 WT cells, normalized to evoked EPSC amplitude at − 70 mV; t-test). **c** Time plot of evoked EPSC amplitude in the presence of the Ca^2+^-permeable AMPAR antagonist, Naspm (50 μM, *n*=7 GluA2^+/ECS(G)^ and 8 WT cells), normalized to the pre-Naspm baseline. Inset: Representative current traces of AMPA EPSCs (recorded at − 70 mV) before and during application of Naspm in WT and GluA2^+/ECS(G)^ mice. HFS induced LTP of fEPSPs in the hippocampal CA1 region of GluA2^+/ECS(G)^ and WT mice, in (**d**) control ACSF (*n*=5 GluA2^+/ECS(G)^ and 7 WT slices; t-test), and in the presence of (**e**) the NMDA receptor antagonist DL-AP5 (100 μM; n=7 GluA2^+/ECS(G)^ and 6 WT slices; t-test), or (**f**) DL-AP5 plus the Ca^2+^-permeable AMPAR antagonist IEM1460 (50 μM; n=5 GluA2^+/ECS(G)^ and 6 WT slices; t-test). In (**d** – **f**) fEPSP slope is normalized over 20 min prior to HFS. **g** Kainate induced Co^2+^ loading in the hippocampus revealed Co^2+^ uptake in the CA1 cell layer of GluA2^+/ECS(G)^ mice. **h** The AMPA and Kainate receptor antagonist NBQX (20 μM), and the non-competitive AMPAR antagonist GYKI 52466 (100 μM) sufficiently blocked Co^2+^ update in the CA1

In the presence of AP5 (50 μM), AMPAR-mediated EPSCs were readily evoked at − 70, 0 and + 40 mV in CA1 neurons from both WT and GluA2^+/ECS(G)^ mice (Fig. 3a and b). While the evoked EPSCs displayed a linear I-V relationship in WT mice, the evoked EPSC I-V relationship displayed inward rectification in GluA2^+/ECS(G)^ mice (Fig. 3a and b). Thus, the normalized evoked EPSC amplitude at + 40 mV was less in GluA2^+/ECS(G)^ compared to WT mice (Fig. 3b; interaction F(2,46) = 18.7, *p < 0.0001*, genotype F(1,23) = 13.7, *p = 0.0012*, voltage F(2,46) = 878, *p < 0.0001*). In addition, AMPAR-mediated evoked EPSCs in GluA2^+/ECS(G)^, but not WT mice, were sensitive to NASPM, a synthetic analogue of Joro spider toxin, which selectively blocks Ca^2+^-permeable AMPARs (Fig. 3c). On average, NASPM (50 μM) inhibited evoked EPSC amplitude by 41 ± 3% in GluA2^+/ECS(G)^ mice, which significantly differed to the 5 ± 4% inhibition observed in WT littermates (Fig. 3c and d; *p < 0.01*).

GluA2-lacking AMPARs are also known to contribute to NMDA receptor-independent LTP, particularly in the CA1 region of the hippocampus [54, 55]. Whether unedited GluA2(Q)-containing AMPARs contribute to NMDA receptor-independent LTP is unknown. High frequency stimulation (HFS) successfully induced LTP in the CA1 region of both GluA2^+/ECS(G)^ mice (180 ± 11% of baseline, *p < 0.05*) and WT mice (123 ± 5% of baseline, *p < 0.05*), but the magnitude of LTP was greater in the GluA2^+/ECS(G)^ mice (*p < 0.001*; Fig. 3d). To determine the contribution of Ca^2+^-permeable AMPARs to LTP, LTP experiments were also performed in slices pre-incubated in the NMDA receptor antagonist DL-AP5 (100 μM). Under these conditions, HFS induced LTP in the CA1 region of GluA2^+/ECS(G)^ mice (144 ± 8% of baseline, p < 0.01), but not WT mice (93 ± 6% of baseline, *p* > 0.05; Fig. 3e). When slices were then pre-incubated in both AP5 (100 μM) and the Ca^2+^-permeable AMPAR antagonist IEM-1460 (50 μM), HFS did not induce LTP in the CA1 region of either GluA2^+/ECS(G)^ mice (96 ± 9% of baseline), or WT mice (105 ± 7% of baseline; Fig. 3f). These results suggest the NMDA-receptor independent LTP observed in GluA2^+/ECS(G)^ mice in the presence of AP5 is dependent on Ca^2+^-permeable AMPARs. This observation is interesting in the context of our earlier observation that GluA2^+/ECS(G)^ mice are vulnerable to NMDAR-independent seizures (Fig. 2c).

Next, we utilised Cobalt (Co^2+^) labelling to directly visualize the presence of Ca^2+^-permeable AMPARs in WT and GluA2^+/ECS(G)^ mice, similar to previous reports [56]. Acute hippocampal slices were stimulated by kainate in the presence of AP5 and TTX. Sparse Co^2+^ staining was observed in the CA3 hippocampal region of both GluA2^+/ECS(G)^ and WT mice (Fig. 3g), presumably in interneurons that are known to express Ca^2+^-permeable AMPARs [57, 58]. In contrast, GluA2^+/ECS(G)^ mice showed enhanced Co^2+^ staining compared to WTs in the CA1 hippocampal region (Fig. 3g). Collectively, these results indicate CA1 neurons exhibit a larger influx of Ca^2+^ (and Co^2+^) than CA3 neurons in GluA2^+/ECS(G)^ mutant mice. To confirm the influx of Co^2+^ was AMPAR mediated, we illustrated that there was little Co^2+^ flux in WT hippocampal slices incubated with AMPAR antagonists NBQX and GYKI, or from GluA2^+/ECS(G)^ mutant hippocampal slices incubated with NBQX, GYKI and Ca^2+^-permeable AMPAR antagonist JSTX (Fig. 3h).

**Fig. 4:**
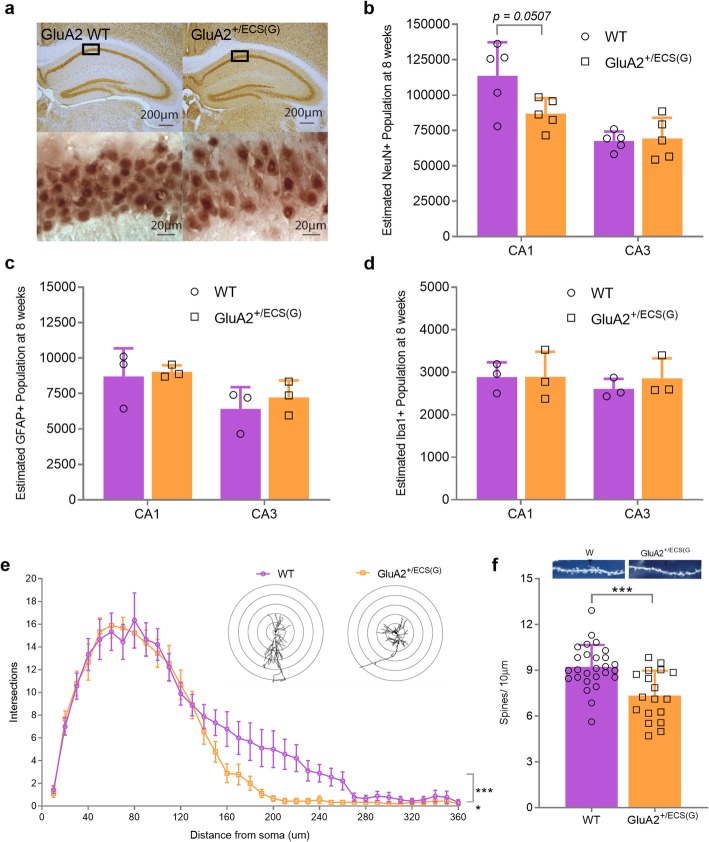
Altered Hippocampal dendritic morphology and neural populations. **a** NeuN+ cells in the hippocampus (10x magnification) and CA1 region (100x magnification) of WT and GluA2^+/ECS(G)^ mice. **b** Cell loss was suggested in the CA1 but not CA3 region of the hippocampus in young adult GluA2^+/ECS(G)^ mice as compared to WT littermates (*n*=5 mice/genotype; unpaired t-test). **c** GFAP+ cell quantification in the CA1 and CA3 of GluA2^+/ECS(G)^ mice as compared to WT littermates (*n*=3 mice/genotype). **d** IBA1+ cell quantification in the CA1 and CA3 of GluA2^+/ECS(G)^ mice as compared to WT littermates (*n*=3 mice/genotype). **e** Inset: Representative traces of CA1 hippocampal neurons from GluA2^+/ECS(G)^ and WT littermates. GluA2^+/ECS(G)^ mice exhibit decreases in dendritic intersections compared to WT controls (*n*=3 neurons/brain, 3 brains/genotype (total 9 neurons/genotype) (two-way ANOVA, * = significant main effect of genotype on distance from soma). **f** Inset: Representative images of CA1 apical dendritic spines from GluA2^+/ECS(G)^ and WT littermates. GluA2^+/ECS(G)^ mice have significantly less spines compared to WT littermates (*n*= 3 dendrites/neuron, 3 neurons/brain, 3 (WT) and 2 (GluA2^+/ECS(G)^) brains/genotype (total 27 (WT) and 18 (GluA2^+/ECS(G)^) apical dendrites/genotype); unpaired t-test). All experiments in Fig. 4 were conducted in 8–10-week-old mice. Data in (**b**), (**c**), (**d**) and (**f**) represent mean ± SD and in (**e**) represent mean ± SEM

Combined, the results presented in Fig. 3 indicate the increased presence of Ca^2+^-permeable AMPARs in GluA2^+/ECS(G)^ mice. The Co^2+^ labelling assay suggests the increased presence of Ca^2+^-permeable receptors is specific to the CA1 region, mirroring the regional specificity of neuron loss in GluA2^+/ECS(G)^ (see Fig. 4b and Additional file 3a). Given subunit assembly does not appear to have been grossly altered in GluA2^+/ECS(G)^ mice (Additional file 2) we suggest the Ca^2+^-permeability of AMPARs in GluA2^+/ECS(G)^ mice is possibly a direct effect of increased unedited GluA2(Q).

### GluA2^+/ECS(G)^ have reduced dendritic density, lower numbers of spines and hippocampal CA1, but not CA3, neuron loss

Importantly, Brusa et al., reported neurodegeneration in the CA3 of juvenile (P20) mice with Q/R site GluA2 editing deficits [37]. Additionally, Feldmeyer et al., reported a reduction in CA3 pyramidal cell dendritic length in P16 mice with reductions in GluA2 Q/R site editing [38]. However, a quantification of synapse, neuronal or glial cell numbers has not yet been conducted on tissue derived from young-adult, or adult mice with genetically reduced levels of GluA2 Q/R site RNA editing. We therefore quantified both CA1 and CA3 neuronal, microglial and astrocyte numbers in young-adult (8–10-week-old) GluA2^+/ECS(G)^ mice. Furthermore, we analysed both dendritic length and/or spine density, specifically in CA1 neurons.

To quantify hippocampal neurons, we used design-based stereology to determine if NeuN+ cell populations were altered in the CA1 and CA3 of GluA2^+/ECS(G)^ mice compared to WT controls (Fig. 4a and b). Intriguingly, we found no differences in NeuN+ numbers in the CA3 of WT vs. GluA2^+/ECS(G)^ mice (Fig. 4b; mean ± SD, t = 0.24, df = 8, *p = 0.82* (unpaired t-test)). In contrast, we observed a strong trend toward cell loss in the CA1 region of GluA2^+/ECS(G)^ mice compared to WT littermates at 8-10 weeks (Fig. 4b; mean ± SD, t = 2.3, df = 8, *p = 0.0507* (unpaired t-test)). In a cohort of GluA2^+/ECS(G)^ mice surviving to 36 weeks we corroborated the CA1 neuron-loss specificity at 8-10 weeks by measuring NeuN+ cell numbers in the CA1 and CA3, finding significant cell loss in the CA1 (Additional file 3a; mean ± SD, t = 5.5, df = 4, *p = 0.0055* (unpaired t-test)). Considering the prior report from Brusa et al., of CA3 damage, the CA1 specificity of neuron loss was unexpected.

Next, we quantified total numbers of IBA1+ microglia and GFAP+ astrocytes in 8-10-week-old mice, finding no significant differences in the numbers of either microglia located within the CA1 or CA3 neuronal cell layer (Fig. 4c; CA1, t = 0.0203, df = 4, *p = 0.9848* (unpaired t-test), CA3, t = 0.8233, df = 4, *p = 0.4566* (unpaired t-test)) or astrocytes located within the CA1 or CA3 cell layer (Fig. 4d; CA1, t = 0.2831, df = 4, *p = 0.7912* (unpaired t-test), CA3, t = 0.7163, df = 4, *p = 0.5134* (unpaired t-test)) in GluA2^+/ECS(G)^ mice compared to WT controls.

Finally, we analysed dendritic length by Sholl analysis of golgi-stained CA1 neurons. We measured this specifically in hippocampal pyramidal CA1 neurons due to the neuron loss in this region (Fig. 4e). Analysis of the main effects illustrated there was an overall significant reduction in the number of dendritic branching points at differing lengths from the neuronal soma in GluA2^+/ECS(G)^ mice compared to WTs (Fig. 4e; interaction F (35, 576) = 1.341, *p = 0.093*, genotype F (1, 576) = 25.22, *p < 0.0001*, distance from soma, F (35, 576) = 62.76, *p < 0.0001* (two-way ANOVA)). Bonferroni *post-hoc* analysis did not reveal any significant differences at individual branch points between the groups. In addition, there was a significant reduction of spine density on CA1 neurons from GluA2^+/ECS(G)^ mice, compared to WT littermates (Fig. 4f; t = 4.1, df = 43, *p = 0.0002* (unpaired t-test)).

Collectively, these results suggest a reduction in GluA2 Q/R site RNA editing leads to hippocampal CA1, but not CA3 neuron loss. The neuron loss does not appear to be associated with a change in the total numbers of microglia and astrocytes at 8-10 weeks, however we note there are many further assessments possible for determining if alterations in microglia and astrocytes may be apparent and possibly contributing to the phenotype of GluA2^+/ECS(G)^ mice, beyond simple cell counts. Furthermore, a GluA2 Q/R site RNA editing deficit appears to lead to a reduction in both the number of dendritic branching points and the total number of spines in CA1 hippocampal pyramidal neurons in vivo.

### GluA2^+/ECS(G)^ have impaired motor function and deficits in fear memory

Although mice with genetically reduced GluA2 Q/R site RNA editing have survived to adulthood in prior studies (with the caveat the average survival is still dramatically reduced [38, 39]), these studies have conducted only brief assessments of motor and cognitive consequences of editing deficits, including open-field behaviour [38] and spatial memory assessments [59] (although the mice in [59] also had a GluA1 KO alongside a GluA2 Q/R site editing deficit). Thus, we assessed exploratory behaviour (open-field test), motor coordination (rotarod) and hippocampal-specific memory (context fear conditioning) of 8-10-week-old GluA2^+/ECS(G)^ mice, compared with WT littermates.

**Fig. 5:**
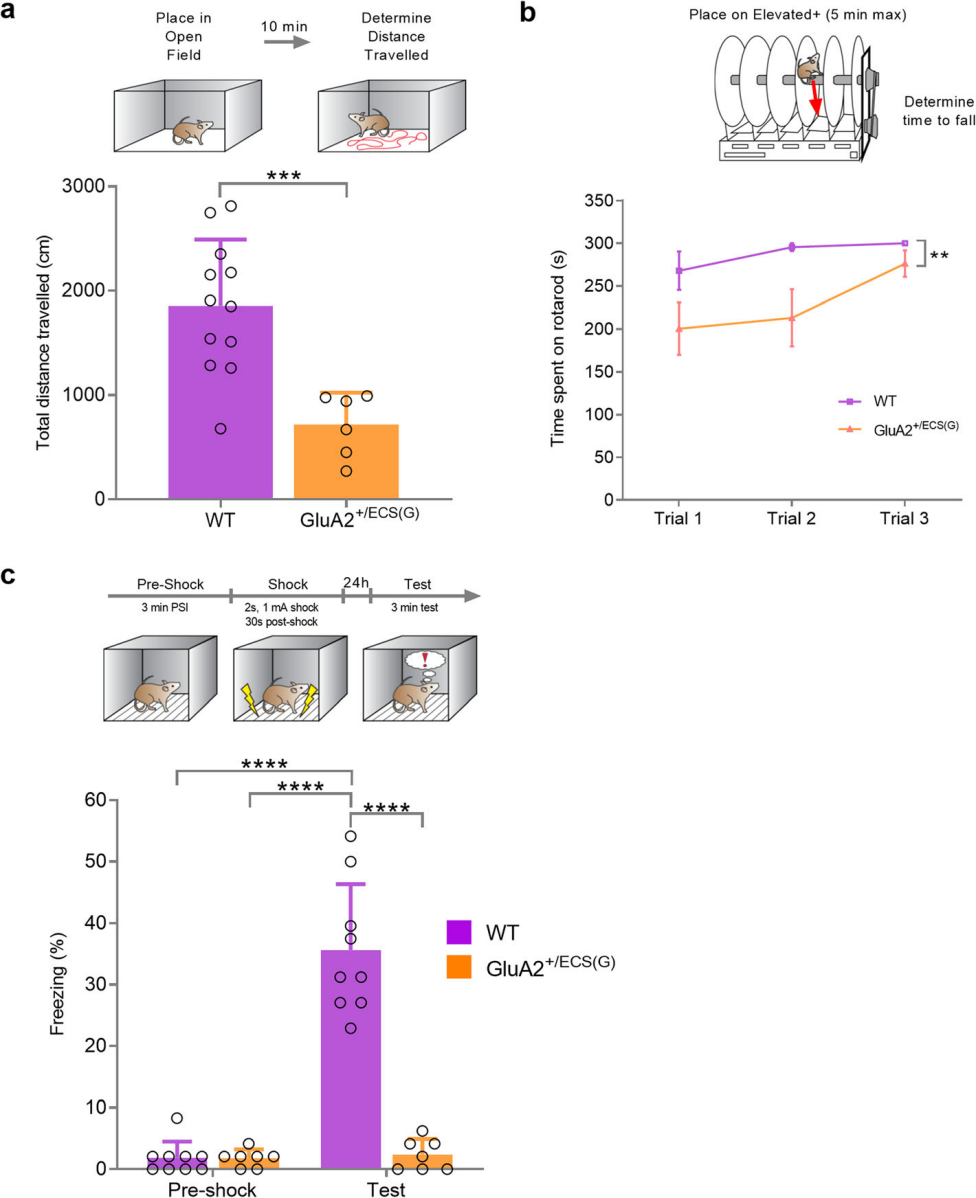
Locomotor, memory and learning deficits in GluA2+/ECS(G) mice. **a** GluA2^+/ECS(G)^ mice exhibit significantly impaired locomotion in the open field test (*n*=12 (WT) and 6 (GluA2^+/ECS(G)^)). **b** GluA2^+/ECS(G)^ mice demonstrate impaired motor coordination and skill learning on the accelerating rotarod over three consecutive trials (*n*=11 in WT and 7 in GluA2^+/ECS(G)^ group; Repeated Measures ANOVA, * = significant main effect of genotype on performance). **c** No significant differences occurred in pre-shock freezing between WT and GluA2^+/ECS(G)^ mice, however GluA2^+/ECS(G)^ mice exhibited significant memory and learning impairments on test day (*n*=9 (WT) and 7 (GluA2^+/ECS(G)^))

In the OFT, GluA2^+/ECS(G)^ mice exhibited a significant reduction in total distance travelled, compared with WT controls (Fig. 5a; mean ± SD, t = 4.12, df = 16, *p = 0.0008* (unpaired t-test)), indicating reduced exploratory behaviour. Furthermore, we observed impaired motor performance in GluA2^+/ECS(G)^ mice, as evidenced by poor performance on the rotarod. Rotarod data from one mouse was removed from the WT group after being identified as a significant outlier using Grubbs’ test. There was both a significant genotype and trial effect, indicating differences between GluA2^+/ECS(G)^ and WT mice that were altered with subsequent trials (Fig. 5b; interaction F(2, 32) = 1.69, *p = 0.2011*, genotype F(1, 16) = 8.86, *p = 0.0090*, trial F(1.9, 29.6) = 5.38, *p = 0.0117* (repeated measures two-way ANOVA with Geisser-Greenhouse correction)).

Next, we assessed hippocampal-specific memory in GluA2^+/ECS(G)^ mice. We conditioned both GluA2^+/ECS(G)^ and WT mice in a context fear conditioning paradigm (pre-Shock and shock, Fig. 5c). 24 h later (Test, Fig. 5c), we re-exposed them to the conditioning context for 3 min in the absence of shock. The magnitude of ‘freezing’ was measured both pre-shock (on Day 1) and during the test (on Day 2) to measure baseline and conditioned fear, respectively. A significant interaction and main effects of both genotype and test session occurred, suggesting that both genotype and test session affected fear, and that the former influenced the latter (Fig. 5c; interaction F(1,28) = 59.45, *p < 0.0001*, genotype F(1,28) = 59.93, *p < 0.0001*, trial F(1,28) = 63.79, *p < 0.0001* (two-way ANOVA)). Bonferroni *post-hoc* analysis revealed no significant differences in pre-shock freezing between genotypes, suggesting no baseline fear alterations in mutant mice. However, during test day, Bonferroni *post-hoc* analysis illustrated WT mice display significantly more freezing than GluA2^+/ECS(G)^ mice (*p < 0.0001*). Furthermore, pre-shock and test freezing did not differ significantly in GluA2^+/ECS(G)^ mice suggesting a deficit in fear expression consistent with impairment of memory acquisition, consolidation and retrieval. Combined, our results reveal severe hippocampal memory deficits and motor coordination in mice that express unedited GluA2 at the Q/R site.

## Discussion

In the present study, we report a new mouse line, called GluA2^+/ECS(G)^, with only a single point mutation in the ECS site on intron 11 of the *Gria2* gene. These GluA2^+/ECS(G)^ mice have reduced GluA2 Q/R site RNA editing, inward rectifying AMPAR currents and altered AMPAR Ca^2+^-permeability, as predicted. They also appear to show grossly normal AMPAR subunit assembly. Meanwhile, the phenotype we observed in the GluA2^+/ECS(G)^ mice was, in general, less severe than that observed in previous models [37, 38]. We discuss our initial observations in these mice below and note that they remain available for future study.

### GluA2^+/ECS(G)^ mice have a GluA2 Q/R site editing deficiency and longer lifespans than previously published models with editing impairments

Three seminal publications have previously characterised genetically modified mice with reduced Q/R site GluA2 RNA editing [37–39]. First, Brusa et al., created a GluA2 Q/R site editing dysfunction by replacing the editing complementary sequence (ECS) and some surrounding DNA within intron 11 of the GluA2 gene (*Gria2*) with a single loxP site [37]. This resulted in mice with ~ 20% of unedited mature cytoplasmic GluA2 mRNA (see [37] for details). Second, Feldmeyer et al., generated several more GM mice with variable levels of editing (98%, ~ 27.8 and 8.7% unedited [38]). In the same publication they also described a transgenic mouse carrying multiple copies of a GluA2 minigene (in addition to endogenous *Gria2* alleles and being expressed in a similar pattern to the endogenous protein) which encoded an asparagine (N) at the Q/R site. Third, Krestel et al., expressed a mutant from Feldmeyer et al., but controlled the expression of this mutation temporally and regionally [39]. In that study, large increases in unedited GluA2(Q) expression were restricted to the forebrain, hippocampal CA1 and dentate gyrus (DG) cells, and could be induced postnatally.

In both Brusa et al., and Feldmeyer et al., the mice generally had dramatically reduced lifespans (<P21) and were prone to spontaneous seizures. In Krestel et al., despite restricting unedited GluA2(Q) expression to postnatal periods, these mice still displayed a high mortality rate (albeit extended compared to constitutive models [38]), with ~ 60% dying from seizures at P60 (~ 8.5 weeks). In contrast to mice created by Brusa et al., and Feldmeyer et al., our constitutive mutant editing deficient mouse exhibited extended survival (although shortened compared with WT mice), with ~ 50% mortality by the age of 9 weeks, but are similarly prone to spontaneous seizures. The mortality rates therefore appear more akin to the model described by Krestel et al. Curiously, the mice from Krestel et al., with forebrain unedited GluA2(Q) expression (restricted to postnatal periods), exhibit a spontaneous seizure phenotype and premature mortality [39], which may suggest seizures are not solely resulting from developmentally generated effects in the various constitutively unedited GluA2 mice.

### GluA2^+/ECS(G)^ mice exhibit altered I/V relations and NMDA receptor independent LTP, concomitant with NMDAR-independent seizure susceptibility

As expected, through the demonstration of inward rectifying current/voltage (I/V) relationships, we identified the presence of Ca^2+^-permeable AMPARs in the CA1 hippocampal region of GluA2^+/ECS(G)^ mice. Our findings are reminiscent of those from previous GluA2 Q/R site editing deficient mouse models [37–39]. The presence of Ca^2+^-permeable AMPARs was further confirmed using Naspm (a selective inhibitor of Ca^2+^-permeable AMPARs). We also demonstrated enhanced LTP in GluA2^+/ECS(G)^ mice that was NMDAR-independent. Thus, Ca^2+^-permeable AMPARs, containing unedited GluA2(Q), might activate pathways normally activated by NMDARs, leading to increased neuronal excitability.

Our results also demonstrate an enhanced KA-induced seizure susceptibility in GluA2^+/ECS(G)^ mice which, as assessed using NMDAR and AMPAR specific inhibitors, can be largely NMDAR-independent. To the best of our knowledge, susceptibility to status epilepticus, induced by KA, has not previously been assessed in mice with constitutively impaired GluA2 Q/R editing. Importantly, AP5, an NMDAR antagonist, has previously been illustrated to have effects in the CNS of rats and mice, when administered peripherally, at equivalent doses to that used here [60–69]. This suggests it is unlikely any lack of effect of AP5 was caused by poor penetration into the brain. Furthermore, we [55] and others [70–72] have previously found CNS effects after peripheral administration of the AMPAR antagonist IEM-1460. We note that it is possible our results may be partially explained by IEM-1460 penetrating the brain more efficiently than AP5. This will be important to rule out in future work.

The results we observed are generally different to those observed in GluA2 knockout or knockdown mice. Hippocampal knockdown of GluA2 in age P13 rats [73], but not adult rats [73, 74] (or adult mice, as we have previously illustrated [55]), leads to an enhanced seizure phenotype in the presence of KA, supporting the concept GluA2 downregulation contributes to seizures in young, but not adult, animals [73]. Meanwhile, intriguingly, GluA2 KO mice have less frequent absence seizures in response to γ-Hydroxybutyric acid than controls [75].

Perhaps most intriguingly, our finding that GluA2^+/ECS(G)^ mice have NMDAR-independent seizures, that are instead dependent on Ca^2+^-permeable AMPARs, may have important clinical implications. Altered RNA editing has been implicated in the aetiology of seizures [76]. Meanwhile, NMDAR antagonists have had mixed results in treating seizures in humans, though they are proposed as second line therapy for status epilepticus [77]. The effect of seizures on GluA2 RNA editing has not been extensively studied and our results suggest the efficacy of NMDAR antagonists may be limited if RNA editing is reduced. Our study provides an imperative to further assess the concept that Ca^2+^-permeable AMPARs, particularly those containing unedited GluA2(Q) subunits, could provide a novel target for seizure control in patients [78, 79].

### GluA2^+/ECS(G)^ mice have CA1 specific neuron loss and CA1 synapse loss

We found neuronal death was specific to the CA1 hippocampal region, with no evidence of degeneration in the CA3 region in adult GluA2^+/ECS(G)^ mice, including in an aged cohort (Additional file 3). The selectivity of the degeneration seems consistent with, and may be explained by, our Cobalt (Co^2+^) labelling results, which suggested a greater concentration of AMPAR-dependent Ca^2+^-permeable cells in the CA1 compared to the CA3 region, a finding that remains open for future investigation.

Our findings contrast to those illustrating dendritic length reduction [38] and cellular [37, 38] degeneration in the CA3 region of previous editing mutant mice and from studies showing a lack of hippocampal degeneration in adult rats following either ADAR2 gene silencing [34], which reduces Q/R site GluA2 editing, or after overexpression of unedited GluA2(Q) in adults [43, 44].

Our results also contrast with a report that unedited GluA2(Q) induces spine growth in pyramidal cells and interneurons in vitro [80], since we found spine and dendritic loss in adult GluA2^+/ECS(G)^ mice. We cannot explain why our findings are different, but it may reflect differences in study design, particularly that our study was in vivo. It is also not yet clear why GluA2^+/ECS(G)^ mice have CA1, but not CA3 neuron loss. CA3 injury and basal dendrite length reductions were previously observed in the young (<P20) GluA2 Q/R editing mutant mice that had prolonged seizure episode and shorter lifespans than GluA2^+/ECS(G)^ mice [37, 38]. Thus, CA3 damage could be explained by the severe seizure phenotype observed in these models. We also note these prior studies did not perform stereological cell counting, as performed in this study and that the design of the mutant mice differs in that these prior models had complete removal of the ECS.

The regional sensitivity to unedited GluA2(Q) could be explained by the higher expression of GluA2 and AMPARs in the CA1, compared with the CA3 and DG [81, 82] and, as suggested above, it is consistent with the finding of greater cobalt staining in the CA1 versus CA3 region. Our finding of CA1 neuron loss is also consistent with studies illustrating unedited GluA2(Q) expression can reduce the threshold for CA1 damage following an acute insult, such as ischemia [34, 43, 44]. In summary, although we cannot completely explain the CA1, but not CA3 neurodegeneration in GluA2^+/ECS(G)^ mice, it is reasonable to consider that increased levels of unedited GluA2(Q) may differentially affect these populations, particularly in the absence of neurotoxic insults.

### GluA2^+/ECS(G)^ mice have impaired motor function and deficits in fear memory

GluA2^+/ECS(G)^ mice exhibited deficits in open field behaviour. The impairment was possibly driven by a reduction in motor coordination, as exhibited by a reduced ability to perform the rotarod task. The observation of motor deficits in GluA2^+/ECS(G)^ mice was not wholly unexpected, not only due to these deficits previously being reported in GluA2 Q/R site editing deficient mice [38] (including in ADAR2 knockdown mice, which also have motor neuron degeneration [83, 84]), but also because of well-established evidence of GluA2 Q/R site editing deficiencies in the spinal motor neurons of sporadic ALS patients [85]. Any future work would benefit from a thorough characterisation of motor deficits, or assessments of motor neuron cell numbers in GluA2^+/ECS(G)^ mice.

The hippocampal fear memory deficit we observed, using the hippocampal-dependent context fear conditioning paradigm, is likely the result of hippocampal synaptic plasticity deficits (Fig. 3), CA1 spine and neuron loss (Fig. 4), or a combination of both. In the context of studies implicating GluA2 Q/R site editing deficits in AD [29, 30], our results provide some evidence to suggest alterations in the proportion of GluA2 Q/R may be capable of aetiologically driving hippocampal learning and memory deficits in dementia, if present in neurons of affected individuals.

We note that GluA2 KO mice have reduced motor coordination on the rotarod [54] and GluA2 lacking receptors can play a role in LTP [54, 86] and NMDAR-independent hippocampal-specific learning [55]. However, we have only undertaken preliminary characterisations of the GluA2^+/ECS(G)^ mice in this study and much more detailed assessments may be valuable in future to tease out the phenotypic differences from GluA2 KOs.

### Limitations

We cannot categorically state, nor do we rule out, that the phenotype of GluA2^+/ECS(G)^ mice may be partially explained by Ca^2+^-signalling through GluA2-lacking AMPARs. However, we consider it unlikely: homomeric unedited GluA2(Q) receptors are known to be delivered to synapses ex vivo [44], recombinant unedited GluA2(Q) containing AMPARs rapidly replace native AMPARs [87], GluA2(Q) homomers are readily trafficked to the cell surface and enable CA1 LTP [88] and unedited GluA2(Q) containing receptors are functional [18–20, 87]. Furthermore, notwithstanding our detection method may not be sensitive enough to detect small changes, GluA2^+/ECS(G)^ mice do not show gross alterations in AMPAR subunit assembly (Additional file 2), in contrast to GluA2 KO mice [49]. We note our preliminary evidence is insufficient to draw strong conclusions, other than a lack of gross changes in receptor assembly.

Additionally, the cell loss we observe in the mice is reminiscent of the effects of unedited GluA2(Q) expression in vitro: viral-mediated expression of unedited GluA2(Q) in primary neurons renders these neurons specifically susceptible to AMPA-induced toxicity, in comparison with cells expressing GluA1 or GluA2(R) [89]. Meanwhile, we, and others, have previously shown GluA2 knockdown or knockout does not lead to hippocampal cell loss [23, 54, 55, 74], unlike that seen in the present study (although we note contrary findings [90, 91]).

We note also that the neurodegeneration we have observed may represent a neurodevelopmental deficit induced by unedited GluA2(Q), a theory supported by findings that overexpression of unedited GluA2(Q) in adult rats does not lead to acute hippocampal neurotoxicity [34, 43, 44]. However, as noted earlier, inducible expression of unedited GluA2 in adult mice does lead to seizures [39], suggesting that the seizure vulnerability is not solely a developmental defect.

Our findings, placed in the context of prior studies illustrating unedited GluA2(Q) containing AMPARs are present at the cell surface, are functional and contribute to AMPAR signalling, make it reasonable to suggest unedited GluA2(Q) incorporation into AMPARs is contributing to the increased AMPAR Ca^2+^-permeability, enhanced NMDAR-independent LTP, CA1 specific-neuron loss and behavioural deficits in GluA2^+/ECS(G)^ mice.

## Conclusions and future directions

The current study provides an initial characterisation of GluA2^+/ECS(G)^ mice. Unedited GluA2(Q) expression could influence GluA2 trafficking [92], maturation or AMPAR tetramerization [50]. At present, we have evidence from GluA2^+/ECS(G)^ mice, provided in Additional file 2, that AMPARs may be forming and trafficking normally, but the data is an initial characterisation only. A comprehensive and sophisticated analysis of gene and protein expression of GluA2, other GluAs, and the myriad of proteins implicated in GluA2 RNA editing, trafficking and AMPAR assembly, as well as trafficking and surface expression analyses, would be of value in future work. Future investigations should also consider unedited and edited GluA2 may have unique regional and temporal effects within different populations of cells and perhaps even within the same population of cells [93, 94].

The observations in the current study provide further support to the idea [34, 84] that unedited GluA2(Q) may be a therapeutically relevant target for preventing neurodegeneration and behavioural impairments in a range of neurological conditions and, given the results of this study, NMDAR-independent seizures. This is, of course, also implied by the reduced GluA2 Q/R site editing efficiency in several neurological conditions including Alzheimer’s disease [29–31], schizophrenia [30], Huntington’s disease [30], amyotrophic lateral sclerosis [32], astrocytoma [33], stroke [34] and cocaine seeking behaviour in rats [35] and by prior observations that overexpression of ADAR2, or overexpression of edited GluA2, can provide therapeutic benefit in some models [34, 35, 84]. GluA2^+/ECS(G)^ mice may therefore offer a new valuable tool for the community going forward and will be made readily available for further study.

## Supplementary information

**Additional file 1:** (A) Q/R site DNA and mRNA sequences. DNA sequencing of the Q/R site revealed the CAG codon in both the WT and GluA2^+/ECS(G)^, indicating no alteration to the site. cDNA sequencing of the site revealed the CGG codon in WT mice, and a marked increase in adenosine in GluA2^+/ECS(G)^ at the CGG codon, confirming the RNA editing assay results in GluA2^+/ECS(G)^ mice. (B) Schematic representation of GluA2 cDNA *BbvI* digestion. The restriction digest produced 2 bands for edited GluA2 (225 bp and 68 bp) and 4 bands for unedited GluA2 (225 bp, 144 bp, 81 bp and 68 bp). (C) *BbvI* restriction enzyme digest of PCR product from WT and GluA2^+/ECS(G)^ mice cDNA. (D) *BbvI* digestion assay results: GluA2^+/ECS(G)^ mice exhibit a significant increase in unedited GluA2 at the Q/R site (*n* = 3/genotype; unpaired t-test).

**Additional file 2:** Schematic representation of the co-immunoprecipitation assay and unbound and bound fraction data. (A) Co-immunoprecipitations were performed by utilising the Dynabead® protein A Immunoprecipitation kit. Dynabead protein A provided was allowed to bind for 20 min with gentle agitation. Following washing, protein sample was added and incubated at RT for 30 min with gentle agitation. The sample was removed via the provided magnet and the unbound fraction (round 1) was kept. The bound fraction was eluted with the provided elution buffer. The unbound fraction was subjected to another round of immunoprecipitations and subsequent to this the fraction (unbound fraction round 2) was used for SDS gel electrophoresis to identify associated AMPA subunits using the appropriate antibody. (B) AMPA receptor subunits remaining after immunoprecipitation of hippocampal homogenates from GluA2^+/ECS(G)^ and WT mice (n = 3/genotype; *t*-test). (C) Co-immunoprecipitation bound fraction analysis revealed correct protein binding. The co-immunoprecipitation bound fraction was a result of one round of precipitation with the Protein A kit, and reveals significant binding of the appropriate proteins in the respective blots. This corresponds to the absence of the respective proteins in B, and shows a correct procedure. The bound fraction cannot be quantified. This is because, as is often the case, two rounds of immunoprecipitation were required to pull down greater than 95%. Lower band represents elution of the antibody.

**Additional file 3:** (A) Significant cell loss in the CA1 but not CA3 region of the hippocampus in 36-week-old adult GluA2^+/ECS(G)^ mice as compared to WT littermates (n = 3/genotype; unpaired t-test). (B) IBA1+ cell quantification in the CA1 and CA3 of 36-week-old GluA2^+/ECS(G)^ mice as compared to WT littermates (n = 3/genotype).

## Data Availability

The datasets used and/or analysed during the current study are available from the corresponding author on reasonable request.

## Abbreviations

ACSF: Artificial cerebrospinal fluid
ADAR: Adenosine deaminase acting on RNA
AMPAR: α-amino-3-hydroxy-5-methyl-4-isoxazolepropionic acid receptor
AP: Anteroposterior
CNS: Central nervous system
DG: Dentate gyrus
ECS: Editing complementary sequence
fEPSP: Field excitatory postsynaptic potentials
HFS: High-frequency stimulation
KA: Kainic acid
LTP: Long-term potentiation
OFT: Open field test
TK: Thymidine kinase
WT: Wild-type
