# Supplementary figures and images for: A new mouse line with reduced GluA2 Q/R site RNA editing exhibits loss of dendritic spines, hippocampal CA1-neuron loss, learning and memory impairments and NMDA receptor-independent seizure vulnerability
Source: Mol Brain. 2020 Feb 27;13:27. doi: 10.1186/s13041-020-0545-1 (PMC7045468; doi:10.1186/s13041-020-0545-1)

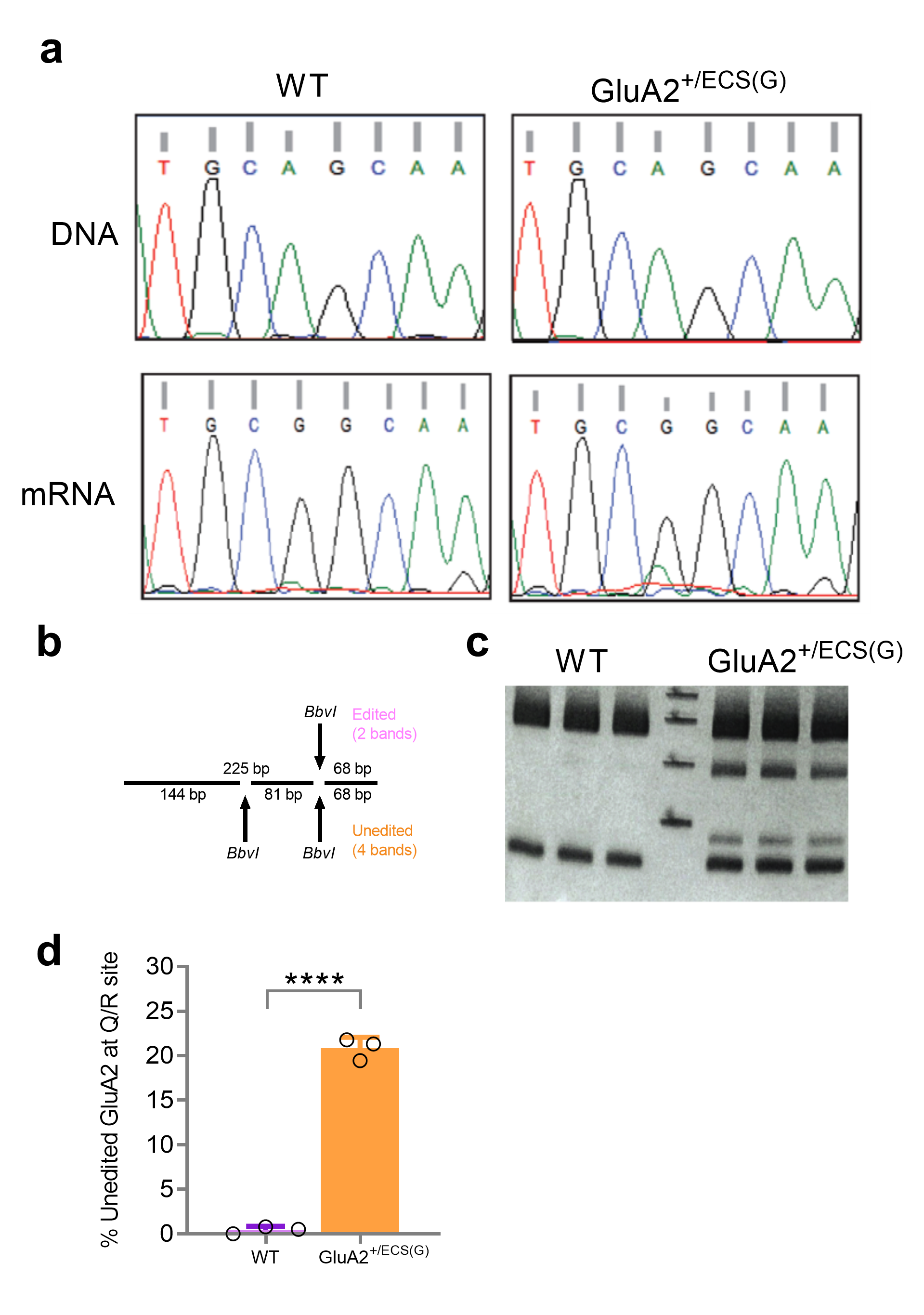

Supplement: Supplementary file 1 — Additional file 1: (A) Q/R site DNA and mRNA sequences. DNA sequencing of the Q/R site revealed the CAG codon in both the WT and GluA2+/ECS(G), indicating no alteration to the site. cDNA sequencing of the site revealed the CGG codon in WT mice, and a marked increase in adenosine in GluA2+/ECS(G) at the CGG codon, confirming the RNA editing assay results in GluA2+/ECS(G) mice. (B) Schematic representation of GluA2 cDNA BbvI digestion. The restriction digest produced 2 bands for edited GluA2 (225 bp and 68 bp) and 4 bands for unedited GluA2 (225 bp, 144 bp, 81 bp and 68 bp). (C) BbvI restriction enzyme digest of PCR product from WT and GluA2+/ECS(G) mice cDNA. (D) BbvI digestion assay results: GluA2+/ECS(G) mice exhibit a significant increase in unedited GluA2 at the Q/R site (n = 3/genotype; unpaired t-test). [file 13041_2020_545_MOESM1_ESM.tif]

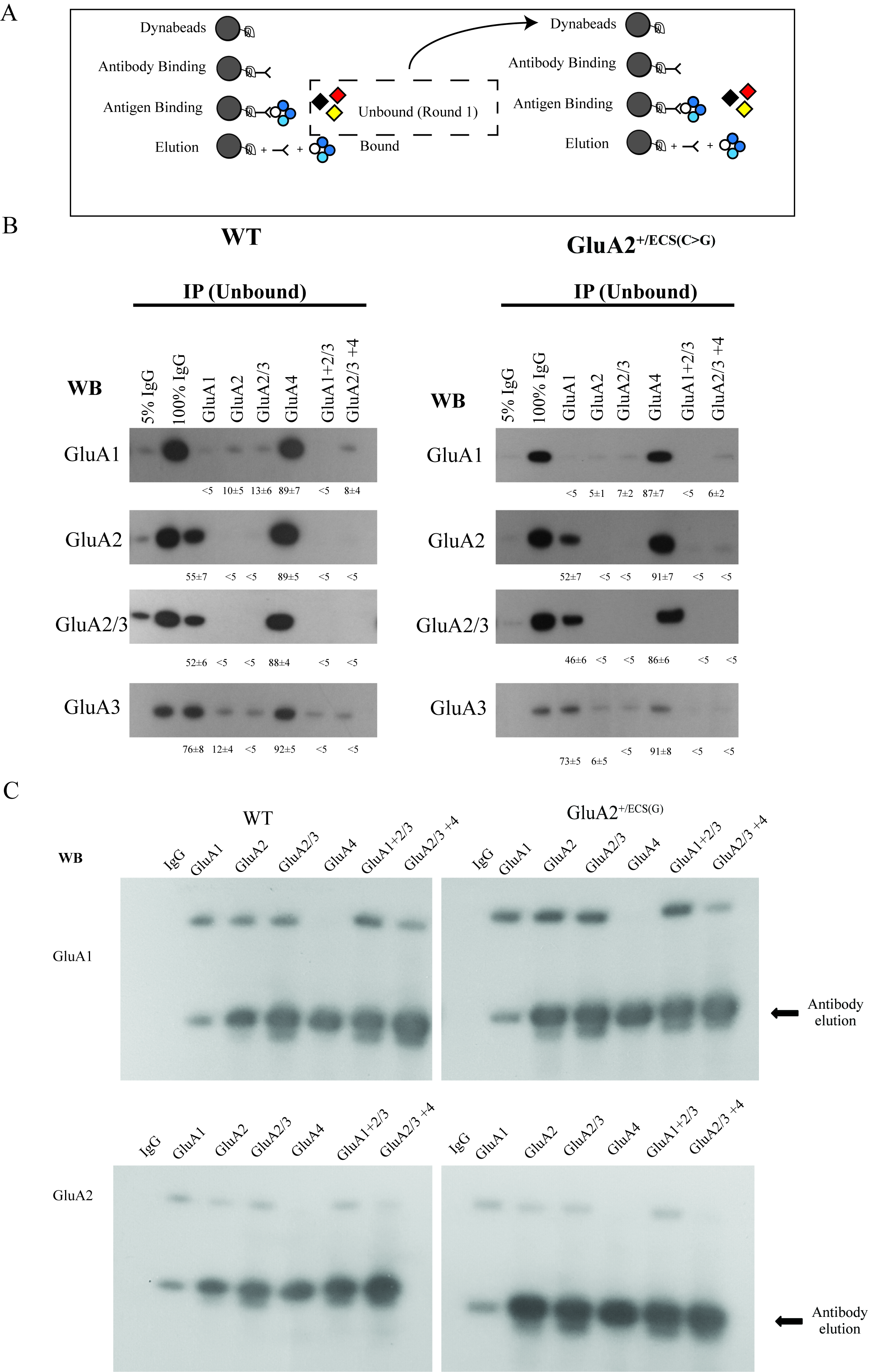

Supplement: Supplementary file 2 — Additional file 2: Schematic representation of the co-immunoprecipitation assay and unbound and bound fraction data. (A) Co-immunoprecipitations were performed by utilising the Dynabead® protein A Immunoprecipitation kit. Dynabead protein A provided was allowed to bind for 20 min with gentle agitation. Following washing, protein sample was added and incubated at RT for 30 min with gentle agitation. The sample was removed via the provided magnet and the unbound fraction (round 1) was kept. The bound fraction was eluted with the provided elution buffer. The unbound fraction was subjected to another round of immunoprecipitations and subsequent to this the fraction (unbound fraction round 2) was used for SDS gel electrophoresis to identify associated AMPA subunits using the appropriate antibody. (B) AMPA receptor subunits remaining after immunoprecipitation of hippocampal homogenates from GluA2+/ECS(G) and WT mice (n = 3/genotype; t-test). (C) Co-immunoprecipitation bound fraction analysis revealed correct protein binding. The co-immunoprecipitation bound fraction was a result of one round of precipitation with the Protein A kit, and reveals significant binding of the appropriate proteins in the respective blots. This corresponds to the absence of the respective proteins in B, and shows a correct procedure. The bound fraction cannot be quantified. This is because, as is often the case, two rounds of immunoprecipitation were required to pull down greater than 95%. Lower band represents elution of the antibody. [file 13041_2020_545_MOESM2_ESM.tif]

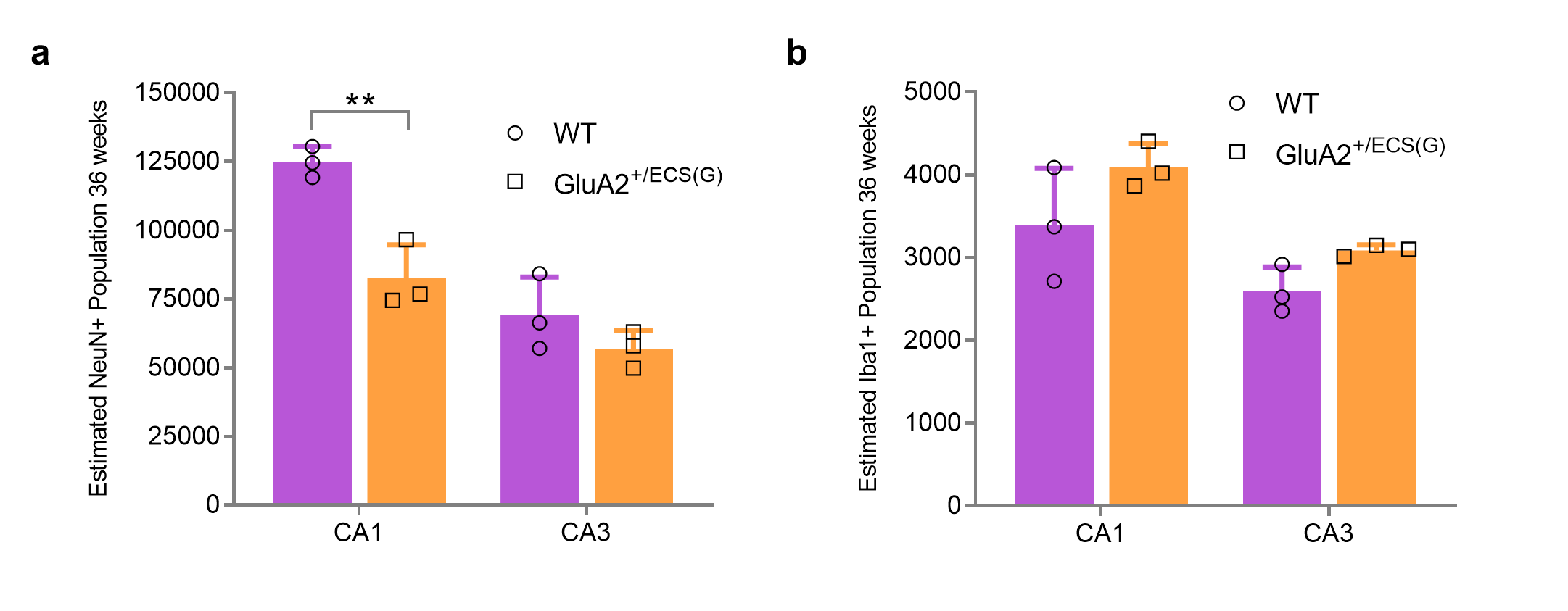

Supplement: Supplementary file 3 — Additional file 3: (A) Significant cell loss in the CA1 but not CA3 region of the hippocampus in 36-week-old adult GluA2+/ECS(G) mice as compared to WT littermates (n = 3/genotype; unpaired t-test). (B) IBA1+ cell quantification in the CA1 and CA3 of 36-week-old GluA2+/ECS(G) mice as compared to WT littermates (n = 3/genotype). [file 13041_2020_545_MOESM3_ESM.tif]
